# Effects of Regular Exercise on the Biochemical, Oxidative, and Inflammatory Profiles and Quality of Life in Older Spaniards with Metabolic Syndrome

**DOI:** 10.3390/antiox13040450

**Published:** 2024-04-11

**Authors:** Margalida Monserrat-Mesquida, Maria Magdalena Quetglas-Llabrés, Cristina Bouzas, Silvia García, David Mateos, Lucía Ugarriza, Cristina Gómez, Josep A. Tur, Antoni Sureda

**Affiliations:** 1 Research Group on Community Nutrition & Oxidative Stress, University of the Balearic Islands-IUNICS, 07122 Palma de Mallorca, Spain; margalida.monserrat@uib.es (M.M.-M.);; 2 CIBEROBN (Physiopathology of Obesity and Nutrition), Instituto de Salud Carlos III, 28029 Madrid, Spain; 3 Health Research Institute of Balearic Islands (IdISBa), 07120 Palma de Mallorca, Spain; 4 C.S. Camp Redó, IBSalut, 07010 Palma de Mallorca, Spain; 5 Clinical Analysis Service, University Hospital Son Espases, 07120 Palma de Mallorca, Spain

**Keywords:** metabolic syndrome, exercise, inflammation, quality of life, adults

## Abstract

Metabolic syndrome increases the risk of developing diabetes and cardiovascular disease. The regular practice of physical activity is a crucial factor for healthy aging and for controlling and preventing chronic diseases. To assess the effects of regular physical activity on the biochemical and inflammatory profiles, as well as the quality of life of older adults diagnosed with metabolic syndrome. Participants (aged 55–70 years; living in the Balearic Islands, Spain) were divided into two groups (*n* = 50 each) according to the degree of physical activity measured by metabolic equivalents of task (METs). Anthropometric parameters, blood pressure, biochemical and hematological parameters, and inflammatory biomarkers were measured. Beck Depression Inventory and adherence to the Mediterranean diet questionnaires, as well as the Dietary Inflammatory Index, chair test, health-related quality of life (HRQoL), and Rapid Assessment of Physical Activity, were also determined. The characterization of the patients was similar in both groups, showing a homogeneous sample. The group with the highest METs experienced a decrease in depression and an increase in the intensity of physical activity. Adherence to the Mediterranean diet and HRQoL physical dimensions increased in participants with the highest METs, also showing a decrease in glycemia and glycosylated hemoglobin values. Inflammatory biomarkers, including tumor necrosis factor alpha, interleukin-6, interleukin-1β, and osteoprotegerin, decreased in patients practicing more physical activity. High levels of physical activity are related to a healthier lifestyle, characterized by high adherence to the Mediterranean diet, decreased depressive behavior, oxidative stress, and inflammatory status in older people with metabolic syndrome.

## 1. Introduction

Metabolic syndrome (MetS) has emerged as a prominent global public health issue due to its increasing prevalence in recent years, affecting approximately 25% of adults worldwide [1]. In Spain, the prevalence is around 22.7% of the older population, and it is anticipated to reach epidemic proportions in the coming years because of its projected exponential growth [2]. MetS is defined as a condition characterized by hyperglycemia, hypertension, hyperlipidemia, hypo-HDL-cholesterolemia, and abdominal obesity [3]. MetS significantly increases the risk of developing metabolic disorders such as type 2 diabetes, cardiovascular diseases, lipid and circulatory disorders (including non-alcoholic fatty liver disease or NAFLD), as well as cancer, neurodegenerative disorders, atherosclerosis, and reproductive issues. Regarding reproductive health, it was pointed out that MetS increased the prevalence of women diagnosed with polycystic ovary syndrome (PCOS) [4]. Moreover, a potential adverse effect of MetS on male reproductive capacity has been observed, with reports indicating a negative correlation between MetS and sperm parameters as well as testosterone levels [5]. MetS also raises the risk of mortality of all causes, making it necessary to develop strategies for early diagnosis and treatment of the underlying risk factors [6]. Especially in elderly individuals, components of MetS are associated with cardiovascular risk (CVR), heart age (HA), and vascular age (VA) [7].

Numerous factors have been recognized to heighten vulnerability to MetS, such as dysfunction in adipose tissue, chronic inflammation, oxidative stress, aging, alterations in microbiota, genetic predisposition, disturbances in circadian rhythm, unhealthy diet, and lack of physical activity [8]. These risks frequently manifest early in life, during childhood and adolescence, and are closely linked to an increased probability of developing chronic diseases later in adulthood. The prevalence of MetS in children ranges from 1.4% in Northwestern Europe to 8.2% in Central Latin America, while in adolescents, it varies between 2.9% in East Asia and 6.7% in high-income English-speaking countries [9]. Despite increasing longevity globally, projections indicate that the proportion of the world’s population over 60 will nearly double, from 12% to 22%, between 2015 and 2050 [10]. The aging of the human population not only poses challenges but also increases the risk of chronic illnesses. As life expectancy rises alongside chronic diseases, susceptibility to frailty, senile dementia, functional decline, and incapacity will also increase, MetS being one of the most common chronic diseases linked to aging [11]. Moreover, central obesity is the predominant cardiometabolic risk factor for MetS in elderly Spanish individuals exhibiting reduced functional capacity [12]. These statistics underscore the global nature of MetS and emphasize the need for comprehensive interventions targeting lifestyle factors and early detection in young and older populations to mitigate the long-term health consequences associated with MetS.

Physical activity, sedentary lifestyle, and sleep are fundamental components of the circadian rhythm, which significantly impacts human health and is associated with multiple cardiometabolic risk factors such as MetS [13,14]. Moreover, depression has been identified as a risk factor for MetS, highlighting the importance of early detection and prevention of depression in the management of MetS [15]. Self-perceived health-related quality of life (HRQoL) could be a robust long-term predictor of chronic illness and mortality [16]. Obesity seems to correlate with a detrimental effect on HRQoL, impacting the physical aspects more significantly than the psychosocial [17]. MetS could potentially reduce HRQoL regarding overall physical well-being, explicitly affecting general health and body pain in men and women, respectively [18].

A low-grade chronic inflammation condition is closely related to metabolic disorders, potentially playing a causative role in the development of insulin resistance, impaired insulin secretion, and disturbances in other aspects of maintaining energy balance [19]. Chronic inflammatory response is marked by irregular production of adipokines and the triggering of pro-inflammatory pathways, leading to various inflammation indicators. The infiltration of macrophages and lymphocytes into adipose tissue plays a role in developing metabolic disorders [20]. Insulin resistance, one of the components of MetS, is frequently noted in older adults. Aging is minutely linked to insulin resistance through low-grade chronic inflammation. This state is marked by the abnormal release of cytokines and the activation of pro-thrombotic and pro-inflammatory pathways, heightening susceptibility to diseases and mortality [21]. Moreover, dyslipidemia, a consequence of MetS, is related to systemic inflammation, representing a critical area of study for improving cardiovascular pathologies, which can improve with anti-inflammatory therapy, alleviating lipid dysfunction [22].

Pro-inflammatory markers, such as tumor necrosis factor α (TNF-α), interleukin-6 (IL-6), interleukin-1β (IL-1β), and leptin, demonstrate a positive correlation with insulin resistance and the characteristics of MetS [23]. Moreover, deregulation of the production of monocyte chemoattractant protein-1 (MCP-1) was observed in MetS patients as a consequence of oxidative stress [24]. An increase in systemic pro-inflammatory and oxidative stress conditions has been observed in MetS patients of both genders when compared to those without MetS [25].

The study hypothesis is that regular exercise has a beneficial effect on biochemical, oxidative stress, and inflammatory profiles, as well as on quality of life in older Spaniards with metabolic syndrome. The aim of the current study was to assess the effects of regular physical activity on the biochemical and inflammatory profiles, and the quality of life of older adults diagnosed with metabolic syndrome.

## 2. Materials and Methods

### 2.1. Study Design

One hundred adults aged 55 to 75 were recruited in the Balearic Islands, Spain, for this study. In order to be eligible for participation in the study, patients were required to satisfy the following criteria: (1) age between 55 and 75 for men and from 60 to 75 for women; (2) body mass index (BMI) between 27 and 40 kg/m^2^; and (3) three or more of the MetS criteria as defined by the updated harmonized criteria from the International Diabetes Federation, National Heart, Lung, and Blood Institute, and the American Heart Association [26]. These criteria encompassed the following aspects: (1) waist circumference exceeding 88 cm for women and 102 cm for men; (2) blood pressure exceeding 130/85 mmHg; (3) fasting serum glucose levels exceeding 100 mg/dL; (4) HDL-cholesterol levels falling below 50 mg/dL in women and 40 mg/dL in men; (5) triglyceride levels exceeding 150 mg/dL.

Participants were categorized into two groups based on their physical activity. Physical activity was evaluated in terms of metabolic equivalents of task (METs) [27], considering the energy expenditure rate in min/day (MET·min/day). The first group comprised low-physical-activity participants (<2240.56 MET·min/day; *n* = 50), and the second group comprised high-physical-activity participants (>2240.56 MET·min/day; *n* = 50).

The experimental protocol adhered to the guidelines outlined in the Declaration of Helsinki and underwent a thorough review and approval process by the Research Ethics Committee of the Balearic Islands (CEICIB2251/14PI). Each participant received a comprehensive explanation of the study’s objectives and potential consequences, and their informed consent was duly obtained.

### 2.2. Anthropometric Parameters

To minimize variations in measurements between different observers, specialized personnel conducted the anthropometric assessments. Body weight was determined using a Segmental Body Composition Analyzer (Tanita BC-418, Tanita, Tokyo, Japan). Measurement was performed with participants wearing light clothing and no shoes, with a deduction of 0.6 kg to account for clothing weight. Height measurements were taken using an anthropometer (Seca 214, SECA Deutschland, Hamburg, Germany) to the nearest millimeter. Body mass index (BMI) was calculated as kg of body weight per m^2^ of height (kg/m^2^).

Waist and hip were measured (cm) using an anthropometric tape; the waist measurement (indicative of abdominal obesity) was taken at the midpoint between the last rib and the iliac crest. The waist-to-hip ratio was calculated.

Blood pressure was measured using a validated semi-automatic oscillometer (Omron HEM, 705CP, Hoofddorp, The Netherlands). Three measurements were obtained after the participant had been seated for 5 min, with a 1 min interval between each determination.

### 2.3. Plasma and Urine Isolation, and Biochemical and Hematological Parameters

Venous blood samples were collected from all participants following a 12 h overnight fast. These samples were collected in ethylene diamine tetra acetic acid (EDTA) sample tubes and subsequently centrifuged at 1700× *g* for 15 min at a temperature of 4 °C. Urine samples were gathered following a 12 h overnight fasting period, taken from the first void of the day, and stored in sterilized containers.

Biochemical parameters, including glucose, glycosylated hemoglobin (HbA1c), triglycerides (TG), high-density lipoprotein cholesterol (HDL-C), total cholesterol, aspartate aminotransferase (AST), alanine aminotransferase (ALT), and gamma-glutamyl transferase (GGT) were determined using established clinical protocols. Hematological parameters such as hematocrit and cell count (erythrocytes, leukocytes, and platelets) were analyzed in whole blood using an automated flow cytometer analyzer (Technion H2, Bayer, VCS system, Frankfurt, Germany).

### 2.4. Quality of Life Parameters

The Beck Depression Inventory II (BDI-II), previously validated, was the assessment tool used to determine the presence of depressive symptoms at both time points [28]. The BDI-II comprises 21 multiple-choice questions, each of which is assigned a singular score. Summing up, these individual scores result in an overall score between 0 and 63. A higher score indicates a greater severity of depressive symptoms.

The evaluation of health-related quality of life (HRQoL) was conducted using a modified edition of the SF-36 HRQoL survey, which had been validated for use in the Spanish population [29]. This survey assesses an individual’s self-perceived HRQoL and categorizes their perceived health across eight different domains: physical functioning, physical role, bodily pain, general health, vitality, social functioning, emotional role, and mental health. In the present study, HRQoL was examined within two overarching categories, physical health, and mental health, which were derived from the amalgamation of the aforementioned eight subdomains [29].

The Dietary Inflammatory Index (DII) evaluates the inflammatory potential of one’s diet [30]. This index considers the impact of 45 different foods, nutrients, and bioactive compounds on six inflammatory biomarkers, including four interleukins (IL-1β, IL-4, IL-6, and IL-10), C-reactive protein (CRP), and tumor necrosis factor alpha (TNF-α). A positive DII score indicates a pro-inflammatory diet, while a negative score suggests an anti-inflammatory diet [30]. The methodology for calculating the DII is detailed elsewhere [30,31]. Each of the 45 dietary components was assigned an inflammatory effect score. The individual’s intake of each component was standardized by subtracting the mean standard intake and dividing by its standard deviation (SD). The resulting centered percentile was then multiplied by the overall inflammatory effect score of the respective dietary component. The sum of these values produced the overall DII score. The impact of the diet on inflammation was categorized as anti-inflammatory when the food score was negative, pro-inflammatory when the score was positive, and having no significant effect on inflammatory biomarkers when the score was zero. This evaluation was conducted based on dietary intake data obtained from a validated FFQ, as previously described [30].

Registered dietitians were responsible for conducting the 17-item MedDiet questionnaire [32] to evaluate compliance with the Mediterranean diet. This questionnaire is a modified adaptation of the validated questionnaire employed in the PREDIMED trial [33]. Each of the 17 items on the questionnaire pertained to wholesome Mediterranean dietary practice. A score of 1 was assigned for adherence to each dietary item, while a score of 0 was given if the item was not adhered to. As a result, the 17-item MedDiet questionnaire yielded a score ranging from 0 to 17, indicating the level of adherence.

Regarding hours of sleep, participants provided information on their average daily sleeping duration for both weekdays and weekends by responding to the open-ended question: “How many hours do you sleep on average per day on weekdays and weekends?”. This question was not validated.

Sedentary lifestyle was measured using the Rapid Assessment of Physical Activity (RAPA), a reliable assessment of physical activity suitable for clinical use with older adults. RAPA is a questionnaire of 9 items, each with ‘yes’ or ‘no’ response options. These questions span the spectrum of physical activity levels, encompassing sedentary behaviors through to vigorous physical activity, as well as strength training and flexibility exercises. The questionnaire’s instructions include a concise description of three activity levels—light, moderate, and vigorous—together with visual and textual representations of activities falling within each category. The cumulative score for the initial seven questions ranges from 1 to 7 points, classifying respondents into five levels of physical activity: 1 = sedentary, 2 = underactive, 3 = regularly underactive (light activities), 4 = regularly underactive, and 5 = regularly active. Responses to the strength training and flexibility queries are evaluated separately, with strength training assigned 1 point, flexibility 2 points, and both activities receiving 3 points [34]. In this study, sedentary (1) and underactive (2) were combined into a new variable, “sedentary or inactive”, and regularly underactive (3) and regularly underactive (4) into the new variable, “moderately active”.

A 30 s chair-stand test previously validated in older adults [35] served as a measure of lower-limb muscle strength. It was administered at the same workstation where dietary assessments and anthropometric measurements were conducted, facilitated by certified physical activity technicians. As previously described, participants’ performance was evaluated based on the number of chair stands completed within the 30 s timeframe [35].

### 2.5. Inflammatory and Oxidative Stress Biomarkers

All immunoassay kits were used with plasma samples, strictly adhering to the manufacturer’s provided instructions for use. Interleukin-1β (IL-1β) and monocyte chemoattractant protein-1 (MCP-1) levels were quantified using dedicated ELISA kits from RayBiotech^®^ (Parkway Lane, Suite, Norcross, GA, USA). TNF-α and xanthine oxidase (XOD) levels were assessed using an ELISA kit from Diaclone (Besançon, France) and an ELISA kit from Cusabio^®^ Technology LLC (Houston, TX, USA), respectively. Lastly, the levels of osteoprotegerin (OPG), interleukin-6 (IL-6), interleukin-15 (IL-15), interleukin-10 (IL-10), resistin, leptin, and ghrelin were assessed utilizing the Human Custom ProcartaPlex^TM^ kit from Invitrogen by Thermo Fisher Scientific (Bender MedSystems GmbH, Vienna, Austria). The concentration of urinary 8-oxo-7,8-dihydro-guanosine (8-oxodG) and 8-oxo-7,8-dihydroguanosine (8-oxoGuo) was assessed using the ultra-performance liquid chromatography coupled with tandem mass spectrometry (UPLC-MS/MS; Waters, Milford, MA, USA) method, as previously outlined [36].

### 2.6. Statistics

Statistical analysis was conducted using the Statistical Package for the Social Sciences (SPSS v.29 for Windows, IBM Software Group, Chicago, IL, USA). Results are shown as mean ± standard deviation (SD), and for all statistical analyses, significance was set at *p* < 0.05. Participants were categorized into two groups according to their physical activity, which was measured in METs. The normal distribution of continuous data was evaluated using histograms and normal probability plots. Statistical significance was assessed using Student’s *t*-test for unpaired data or with the Mann–Whitney U test, depending on the data distribution. Categorical variables were expressed as a percentage and analyzed using the chi-square (χ^2^) test. Logistic regression analysis was conducted to estimate the odds ratio (OR) and the corresponding 95% confidence interval (CI). This analysis aimed to assess the relationship between inflammatory and oxidative biomarkers and physical activity. Low physical activity was established as a reference. Logistic regression analysis was adjusted for age, sex, body mass index (BMI), and waist-to-hip ratio (WHR).

## 3. Results

The findings were organized into two groups based on the physical activity of participants. The first group consisted of individuals with low physical activity (<2240.56 MET·min), and the second group, those with high physical activity (>2240.56 MET·min/day).

### 3.1. Anthropometric and Hematological Parameters

The anthropometric and biochemical parameters classified according to physical activity are shown in Table 1. The participant characteristics were similar in both groups, with no significant differences reported, indicating a homogeneous sample in this study. Participants with high levels of physical activity showed lower glycemia and glycosylated hemoglobin levels. Table 2 shows the results from hematological parameters; participants with high levels of physical activity showed a lower number of leukocytes than those with low physical activity.

### 3.2. Quality of Life Parameters

Quality of life parameters according to physical activity are shown in Table 3. In the high physical activity group, there was an increase in the results of the 30 s chair stand test, adherence to the Mediterranean diet, and HRQoL physical dimensions; depression levels and inflammatory diet scores were reduced.

### 3.3. Inflammatory Biomarkers

Plasma levels of inflammatory and oxidative stress biomarkers are represented in Table 4 according to the physical activity of participants. Significant reductions were observed in plasma levels of TNF-α, OPG, IL-1β, and IL-6 in participants with the highest physical activity. In urine samples, 8-oxoGuo showed a significant reduction in the highest physical activity group. No differences were observed in other biomarkers. Table 5 shows crude and adjusted ORs for the association between inflammatory and oxidative biomarkers and physical activity in participants with metabolic syndrome. Low physical activity was established as a reference. Crude and adjusted OR analysis showed that high physical activity was considered a protective factor against high glycemia levels and inflammatory levels of TNF-α, IL-1β, and IL-6. Physical activity also seemed to be a protective factor for OPG levels.

## 4. Discussion

This research shows an association between high physical activity, oxidative stress, and pro-inflammatory biomarkers. The study participants with higher physical activity also showed better quality of life parameters, suggesting the potential for promoting healthier lifestyles among patients with MetS. The participants in the study constituted a homogeneous population from the Balearic Islands with MetS, and consequently, no significant differences were observed in patient characteristics. Therefore, the results may not fully reflect the outcomes in a more diverse group of people.

The beneficial effects of physical activity are in accordance with previous evidence [37,38], which pointed out that physical activity has clinical implications, particularly the reduction in cardiovascular risk factors. Combined exercise was shown to be the best for improving weight, waist circumference, diastolic blood pressure, triglycerides, glycemia, and insulin levels [37]. Additionally, interventions promoting exercise and healthy lifestyle behaviors have shown positive effects on cardiometabolic risk indicators among patients with MetS [38]. A noteworthy finding from our study is the significant reduction in fasting glycemia and glycosylated hemoglobin levels observed in participants with high physical activity. This reduction could mitigate cardiovascular risk factors, as supported by previous findings indicating higher levels of fasting glycemia and glycosylated hemoglobin in patients with diabetes mellitus [39].

MET is an indicator of the intensity of physical activity [40]. It was observed that leisure-time physical activity was associated with a decrease in the risk of suffering MetS [41,42]. Previous findings showed that the lack of physical activity increases the severity of MetS, as well as sedentary time, depression risk, and low adherence to the Mediterranean diet [43].

Current high physical activity is related to high levels of chair tests, adherence to the Mediterranean diet, as well as low levels of DII and risk of depression. These current results indicate that lifestyle improvements associated with physical activity, as well as the promotion of physical activity and healthy behaviors, are associated with a decrease in the severity of MetS [44,45]. It has been observed that a reduction in MetS severity is related to high leisure-time physical activity, high adherence to the Mediterranean diet, anti-inflammatory dietary patterns, low sedentary time, and low depression risk [44]. Moreover, this current study revealed that patients with higher physical activity levels exhibited improved physical dimensions of HRQoL. Accordingly, it has been observed that individuals with poor cardio-metabolic health and low levels of activity should be a crucial focus for promoting health and well-being. Encouraging physical activity during the early stages of aging appears crucial to reducing the effects of MetS on HRQoL as individuals age [46]. The quality of life parameters of this current study are in accordance with those of a previous study, which observed that personalized aerobic training over 8 weeks yielded a beneficial impact on the subjects with MetS, improving their HRQOL, motivation for physical activity, and reducing levels of depression [47].

The current study also showed a significant improvement in pro-inflammatory and oxidative stress status in patients with higher physical activity. These findings indicate a reduction in the MetS risk profile, as previously observed; however, exercise also has anti-inflammatory effects, reducing high-sensitivity C-reactive protein and IL-6 levels in patients with MetS [48]. Isolated aerobic exercise, combined with aerobic and resistance exercise, also seems to be optimal for improving cytokine levels, such as TNF-α, IL-8, and IL-10 levels, in patients with MetS [49]. In this sense, the significant reduction in IL-1β signaling in the group with high physical activity indicates a reduction in chronic inflammation associated with MetS. Deregulated IL-1β levels can result in severe acute or chronic inflammation, leading to devastating diseases [50].

OPG is a glycoprotein that plays a role in bone metabolism, regulating functions in the immune, skeletal, and vascular systems. Circulating levels of OPG have been identified as biomarkers for cardiovascular disease in both patients with acute or chronic heart conditions and in healthy individuals. OPG has been associated with different types of inflammation and has been linked to diabetes along with inadequate glycemic management [51]. Therefore, a significant reduction in OPG among MetS participants with higher physical activity shows that inflammation was reduced, and glycemic status improved.

Increased nucleoside damage generated by oxidative stress, measured by 8-oxodG to DNA and 8-oxoGuo to RNA, was linked to higher cardiovascular risk scores and higher levels of insulin resistance [52]. Thus, the significantly reduced levels of 8-OxoGuo observed in this current study in patients with high physical activity could show a reduction in cardiovascular risk in patients with MetS.

### Strengths and Limitations

The main strength of this current research is the association between high physical activity and improvement in oxidative stress and inflammatory status, and the quality of life among patients with MetS. However, the first limitation of this current research is the relatively small sample size; despite this drawback, it is important to note that the sample size was adequate to identify noticeable differences in inflammatory biomarkers between participants with high physical activity and those with low physical activity. Secondly, current participants were between 55 and 75 years old. Therefore, the findings and conclusions of the present study may not directly apply to a younger population and cannot be generalized. Hence, future research should consider these age-related variations when attempting to extend these findings to a broader and more diverse demographic. Finally, causal inferences cannot be established because of the cross-sectional design, and prospective analysis should be developed in future research.

## 5. Conclusions

High levels of physical activity are related to a healthier lifestyle, characterized by high adherence to the Mediterranean diet, decreased depressive behavior, oxidative stress, and inflammatory status in older people with metabolic syndrome. Therefore, it is recommended to promote regular physical activity among patients with metabolic syndrome to improve their healthy lifestyle, which could improve public health.

## Figures and Tables

**Table 1:** Characterization of participants with metabolic syndrome according to physical activity.

	Low Physical Activity *n* = 50	High Physical Activity *n* = 50	*p*-Value
	Mean (SD)	Mean (SD)	
Weight (kg)	88.0 (14.5)	87.3 (12.8)	0.661
Waist (cm)	111.2 (10.2)	111.2 (10.0)	0.984
Hip (cm)	113.7 (10.1)	111.9 (8.6)	0.116
Height (cm)	162.8 (9.3)	162.9 (9.3)	0.945
BMI (kg/m^2^)	33.1 (3.7)	32.8 (3.5)	0.574
Systolic blood pressure (mmHg)	140.6 (19.4)	140.2 (16.3)	0.834
Diastolic blood pressure (mmHg)	82.1 (11.0)	80.9 (10.4)	0.358
Glycemia (mg/dL)	124.4 (44.8)	111.7 (22.4)	0.003
HbA1c (%)	6.45 (1.4)	5.99 (0.75)	0.001
Triglycerides (mg/dL)	155.2 (79.0)	147.4 (64.3)	0.377
HDL-cholesterol (mg/dL)	43.7 (10.2)	44.7 (10.6)	0.447
Total cholesterol (mg/dL)	185.3 (38.9)	184.4 (34.1)	0.842
AST (U/L)	20.5 (6.9)	20.7 (5.4)	0.783
ALT (U/L)	22.1 (9.3)	21.9 (8.4)	0.878
GGT (U/L)	34.2 (27.8)	30.4 (16.4)	0.191
Physical activity (MET·min/day)	1023.3 (708.8)	4743.1 (2870.1)	<0.001

Abbreviations: BMI, body mass index; Hb1Ac, glycated hemoglobin 1A; HDL-cholesterol, high-density lipoprotein; AST, aspartate aminotransferase; ALT, alanine aminotransferase; GGT, gamma glutamyl transferase; SD, standard deviation. *p*-values by Student’s *t*-test.

**Table 2:** Hematological parameters of participants with metabolic syndrome according to physical activity.

	Low Physical Activity *n* = 50	High physical Activity *n* = 50	*p*-Value
	Mean (SD)	Mean (SD)	
Hematocrit (%)	43.0 (4.0)	43.0 (4.1)	0.952
Erythrocytes (10^6^/mm^3^)	4.80 (0.5)	4.78 (0.5)	0.683
Leukocytes (10^3^/mm^3^)	7.59 (1.9)	7.12 (1.6)	0.030
Neutrophils (10^3^/mm^3^)	4.75 (4.8)	4.00 (1.6)	0.083
Lymphocytes (10^3^/mm^3)^	2.46 (0.8)	2.54 (2.3)	0.710
Monocytes (10^3^/mm^3^)	0.64 (0.2)	0.62 (0.2)	0.389
Eosinophils (10^3^/mm^3^)	0.30 (0.6)	0.23 (0.2)	0.174
Basophils (10^3^/mm^3^)	0.095 (0.4)	0.046 (0.03)	0.131

Abbreviations: SD, standard deviation. *p*-values by Student’s *t*-test.

**Table 3:** Quality of life parameters from participants with metabolic syndrome according to physical activity.

	Low Physical Activity *n* = 50	High Physical Activity *n* = 50	*p*-Value
	Mean (SD)	Mean (SD)	
Hours of sleep	7.22 (1.3)	7.31 (1.3)	0.568
Chair test	11.3 (4.6)	12.6 (4.4)	0.027
BDI-II	7.81 (6.7)	5.27 (5.1)	0.001
AMD	6.92 (2.2)	8.07 (2.6)	<0.001
DII	0.681 (2.1)	−0.148 (2.0)	0.003
HRQoL physical dimensions	45.5 (8.3)	48.0 (8.0)	0.039
HRQoL mental dimensions	48.8 (10.8)	50.1 (10.6)	0.437
	*n* (%)	*n* (%)	
RAPA			<0.001
Sedentary or inactive	40 (79.9)	23 (45.9)	
Moderately active	7 (14.9)	14 (28.1)	
Active	3 (5.2)	13 (25.9)	

Abbreviations: BDI-II, Beck Depression Inventory II; AMD, adherence to Mediterranean diet; DII, Dietary Inflammatory Index; HRQoL, health-related quality of life; RAPA, Rapid Assessment of Physical Activity; SD, standard deviation. *p*-values by Student’s *t*-test and X^2^.

**Table 4:** Inflammatory plasma markers and oxidative stress biomarkers of participants according to physical activity.

	Low Physical Activity *n* = 50	High Physical Activity *n* = 50	*p*-Value
	Mean (SD)	Mean (SD)	
MCP1 (pg/mL)	236.3 (87.5)	231.3 (83.9)	0.790
TNF-α (pg/mL)	4.42 (1.9)	2.99 (1.6)	0.003
OPG (pg/mL)	34.3 (15.3)	19.4 (11.9)	<0.001
IL-1β (pg/mL)	12.6 (7.2)	8.96 (4.0)	0.003
IL-6 (pg/mL)	7.49 (5.1)	3.22 (2.3)	0.002
IL-10 (pg/mL)	1.33 (0.7)	1.08 (0.2)	0.310
IL-15 (pg/mL)	6.86 (3.8)	8.06 (4.8)	0.327
Resistin (ng/mL)	7.17 (8.9)	3.35 (1.8)	0.025
Leptin (ng/mL)	11.03 (8.6)	11.9 (14.7)	0.815
Ghrelin (pg/mL)	307.7 (57.4)	289.6 (51.2)	0.212
8-OxoGuo/Creatinine (nM/mM)	2.15 (0.6)	1.86 (0.4)	0.006
8-oxodG/Creatinine (nM/mM)	1.51 (0.6)	1.39 (0.7)	0.322

Abbreviations: MCP1, monocyte chemoattractant protein-1; TNF-α, tumor necrosis factor alpha; OPG, osteoprotegerin; IL-1β, interleukin-1β; IL-6, interleukin-6; IL-10, interleukin-10; IL-15, interleukin-15; 8-oxoGuo, 8-oxo-7,8-dihydroguanosine; 8-oxodG, 8-oxo-7,8-dihydro-guanosine; SD, standard deviation. *p*-values by Student’s *t*-test and X^2^.

**Table 5:** Association between inflammatory and oxidative biomarkers and physical activity in participants with metabolic syndrome.

		Low Physical Activity *n* = 50	High Physical Activity *n* = 50	*p*-Value
		OR (95% CI)	OR (95% CI)	
Cholesterol	Crude OR	1.00 (ref.)	1.219 (0.751–1.978)	0.423
	Adjusted OR	1.00 (ref.)	1.366 (0.828–2.252)	0.222
Triglycerides	Crude OR	1.00 (ref.)	0.986 (0.610–1.593)	0.954
	Adjusted OR	1.00 (ref.)	0.964 (0.591–1.573)	0.883
Glycemia	Crude OR	1.00 (ref.)	0.640 (0.396–1.034)	0.068
	Adjusted OR	1.00 (ref.)	0.579 (0.352–0.951)	0.031
Systolic blood pressure	Crude OR	1.00 (ref.)	1.045 (0.648–1.686)	0.856
	Adjusted OR	1.00 (ref.)	1.045 (0.644–1.697)	0.857
Diastolic blood pressure	Crude OR	1.00 (ref.)	0.648 (0.401–1.048)	0.077
	Adjusted OR	1.00 (ref.)	0.612 (0.371–1.007)	0.053
MCP1	Crude OR	1.00 (ref.)	0.874 (0.367–2.079)	0.761
	Adjusted OR	1.00 (ref.)	0.757 (0.298–1.922)	0.558
TNF-α	Crude OR	1.00 (ref.)	0.295 (0.99–0.881)	0.029
	Adjusted OR	1.00 (ref.)	0.258 (0.076–0.878)	0.030
OPG	Crude OR	1.00 (ref.)	0.160 (0.050–0.512)	0.002
	Adjusted OR	1.00 (ref.)	0.164 (0.049–0.552)	0.004
IL-1β	Crude OR	1.00 (ref.)	0.338 (0.140–0.814)	0.016
	Adjusted OR	1.00 (ref.)	0.266 (0.102–0.691)	0.007
IL-6	Crude OR	1.00 (ref.)	0.208 (0.052–0.830)	0.026
	Adjusted OR	1.00 (ref.)	0.172 (0.034–0.877)	0.034
IL-10	Crude OR	1.00 (ref.)	1.000 (0.167–5.985)	1.000
	Adjusted OR	1.00 (ref.)	0.408 (0.028–6.013)	0.514
IL-15	Crude OR	1.00 (ref.)	1.058 (0.350–3.193)	0.921
	Adjusted OR	1.00 (ref.)	1.021 (0.280–3.719)	0.975
Resistin	Crude OR	1.00 (ref.)	0.601 (0.206–1.752)	0.351
	Adjusted OR	1.00 (ref.)	0.611 (0.197–1.896)	0.394
Leptin	Crude OR	1.00 (ref.)	0.701 (0.218–2.261)	0.552
	Adjusted OR	1.00 (ref.)	0.763 (0.211–2.759)	0.680
Ghrelin	Crude OR	1.00 (ref.)	0.505 (0.177–1.441)	0.202
	Adjusted OR	1.00 (ref.)	0.557 (0.175–1.777)	0.323
8-OxoGuo/Creatinine	Crude OR	1.00 (ref.)	0.585 (0.277–1.236)	0.160
	Adjusted OR	1.00 (ref.)	0.601 (0.277–1.303)	0.197
8-oxodG/Creatinine	Crude OR	1.00 (ref.)	0.586 (0.284–1.209)	0.148
	Adjusted OR	1.00 (ref.)	0.577 (0.272–1.223)	0.152

Abbreviations: OR, odds ratio; Adjusted OR, odds ratio after adjustments for sex, age, body mass index (BMI), and waist-to-hip-ratio (WHR); ref., reference; TNF-α, tumor necrosis factor alpha; OPG, osteoprotegerin; IL-1β, interleukin-1β; IL-6, interleukin-6; IL-10, interleukin-10; IL-15, interleukin-15; 8-oxoGuo, 8-oxo-7,8-dihydroguanosine; 8-oxodG, 8-oxo-7,8-dihydro-guanosine.

## Data Availability

There are restrictions on the availability of the data from this trial due to the signed consent agreements around data sharing. Researchers wishing to access the trial data used in this study can request access from the corresponding author: pep.tur@uib.es.
